# Emergent Prophylactic, Reparative and Restorative Brain Interventions for Infants Born Preterm With Cerebral Palsy

**DOI:** 10.3389/fphys.2019.00015

**Published:** 2019-01-28

**Authors:** Megan Finch-Edmondson, Catherine Morgan, Rod W. Hunt, Iona Novak

**Affiliations:** ^1^The Discipline of Child and Adolescent Health, The Children's Hospital at Westmead Clinical School, The University of Sydney Medical School, Sydney, NSW, Australia; ^2^Cerebral Palsy Alliance Research Institute, The University of Sydney, Sydney, NSW, Australia; ^3^Department of Neonatal Medicine, The Royal Children's Hospital, Melbourne, VIC, Australia; ^4^Department of Paediatrics, University of Melbourne, Melbourne, VIC, Australia; ^5^Neonatal Research, Murdoch Children's Research Institute, Melbourne, VIC, Australia; ^6^Department of Obstetrics and Gynaecology, Monash University, Melbourne, VIC, Australia

**Keywords:** preterm, brain injury, cerebral palsy, neuroplasticity, neuroprotection, neuro-regeneration, neuro-repair

## Abstract

Worldwide, an estimated 15 million babies are born preterm (<37 weeks' gestation) every year. Despite significant improvements in survival rates, preterm infants often face a lifetime of neurodevelopmental disability including cognitive, behavioral, and motor impairments. Indeed, prematurity remains the largest risk factor for the development of cerebral palsy. The developing brain of the preterm infant is particularly fragile; preterm babies exhibit varying severities of cerebral palsy arising from reductions in both cerebral white and gray matter volumes, as well as altered brain microstructure and connectivity. Current intensive care therapies aim to optimize cardiovascular and respiratory function to protect the brain from injury by preserving oxygenation and blood flow. If a brain injury does occur, definitive diagnosis of cerebral palsy in the first few hours and weeks of life is difficult, especially when the lesions are subtle and not apparent on cranial ultrasound. However, early diagnosis of mildly affected infants is critical, because these are the patients most likely to respond to emergent treatments inducing neuroplasticity via high-intensity motor training programs and regenerative therapies involving stem cells. A current controversy is whether to test universal treatment in all infants at risk of brain injury, accepting that some patients never required treatment, because the perceived potential benefits outweigh the risk of harm. Versus, waiting for a diagnosis before commencing targeted treatment for infants with a brain injury, and potentially missing the therapeutic window. In this review, we discuss the emerging prophylactic, reparative, and restorative brain interventions for infants born preterm, who are at high risk of developing cerebral palsy. We examine the current evidence, considering the timing of the intervention with relation to the proposed mechanism/s of action. Finally, we consider the development of novel markers of preterm brain injury, which will undoubtedly lead to improved diagnostic and prognostic capability, and more accurate instruments to assess the efficacy of emerging interventions for this most vulnerable group of infants.

## Prematurity

Prematurity remains the leading cause of morbidity and mortality in childhood within the developed world. Global rates of prematurity range from 5 to 18% of all births (Blencowe et al., 2012). In 2015, 9% of all Australian births occurred <37 weeks' gestational age, giving a burden of prematurity of around 27,000 babies (AIHW, 2017). The incidence of babies born extremely prematurely (22–27^+6^ weeks' gestation) is lower, however over 3,000 Australian babies were born very preterm (<30 weeks' gestation) during the same period (AIHW, 2017). Advances in neonatal intensive care ensure that 85% of these very preterm infants survive, but 55% survive with neurobehavioral impairments, 22% with intellectual disability and 7% with cerebral palsy (Aarnoudse-Moens et al., 2009). In the long term it is estimated that their lifetime cost to the health economy will exceed $200 million (Access Economics, 2008).

### Current Usual Care for Preterm Infants

Usual care of the preterm infant in the Neonatal Intensive Care Unit (NICU) environment is generally highly protocolized and whilst some variation in practice exists between units, the basic “recipe” is the same (Malcolm, 2014). Almost without exception preterm infants with surfactant deficiency receive exogenous surfactant, with the expectation that reduced severity of lung disease will minimize hypoxia and fluctuations in blood pressure (Polin and Carlo, 2014). Oxygen saturations are targeted to around 90–95%, with a recent increase in our understanding that both hypoxia and hyperoxia increase the risk of cerebral and other end organ injury (Askie et al., 2017). Mean blood pressure is targeted to the gestational age at birth in weeks, and fluctuations in blood pressure are avoided where possible, through judicious use of fluid following assessment of fluid status by clinical indicators such as urine output, perfusion and in some centers, ultrasound measures (Kenner et al., 1998). Where there is any risk of infection/inflammation, there is generally a low threshold for the use of broad spectrum antibiotics to cover against the common bacteria that pose a risk to the preterm infant, most notably Group B streptococcus and *Escherichia coli* (Simonsen et al., 2014). The most premature and smallest infants are at most risk of organ damage, however a recent study has demonstrated that even those infants born in the late premature phase are at risk of neurodevelopmental impairments (Cheong et al., 2017). The goal of all of these therapies collectively is to maintain adequate and steady perfusion and oxygenation of the brain to avoid inflammation and brain injury, but none specifically target either neuroprotection or neural repair.

### Brain Injury in Preterm Infants

#### Brain Injury Diagnostics

Historically, cerebral injury during the perinatal period until term corrected age was detected with cranial ultrasound (cUS). However, the sensitivity and specificity of this imaging modality is variable between operators. cUS remains in common use because of its clinical utility with imaging possible at the bedside, and lower costs compared to magnetic resonance imaging (MRI). With cUS, it is possible to detect common patterns of severe preterm brain injury, such as intraventricular hemorrhage (IVH) and periventricular leukomalacia (PVL), however subtle lesions can be indiscernible (Hintz et al., 2007). Serial cUS is therefore widely used for screening, however approximately one third of preterm infants diagnosed with cerebral palsy may not have lesions identified by cUS (Beaino et al., 2010). The accuracy of cUS to predict long term disability is enhanced when scans are taken sequentially until term-equivalent age, and are then combined with MRI to detect and classify the presence of white matter injury (Martinez-Biarge et al., 2016). MRI provides better anatomical detail and has the sensitivity to detect subtle white matter injury that may not be discernible on ultrasound. However, in experienced hands cUS can still be reliable in the detection of cerebral injury, and whilst MRI provides modest benefit to injury detection (Leijser et al., 2008; Edwards et al., 2018), its cost and availability preclude its routine use in some centers.

#### White Matter Injury

With increasing use of MRI, a more insidious cerebral insult—*diffuse white matter injury* has been recognized. Damage to the white matter is the most common type of preterm brain injury with severity ranging from mild to severe (Inder et al., 2003). In white matter injury, the preoligodendrocytes are “arrested” in their development, preventing full maturation and thus myelination (Ferriero, 2016). In addition, recurrent infection and long term ventilation exacerbate neuronal and axonal damage (Khwaja and Volpe, 2008), and results in a higher likelihood of white matter injury (Smilga et al., 2018).

Cystic PVL is a condition in which cystic cavitations develop in the periventricular white matter, and their extent can be graded from grade 1 to 3 (classification by De Vries et al., 1992). PVL has been associated with spastic cerebral palsy, with severity of impairment influenced by the location and extent of the lesions (De Vries et al., 2011). With increasing use of MRI and detection of more subtle white matter injury, the term PVL has been applied to non-cystic diffuse white matter injury (Khwaja and Volpe, 2008). Non-cystic PVL is characterized by microscopic focal necroses which generally evolve to form glial scars (Khwaja and Volpe, 2008). The prevalence of cystic PVL has been declining for some time (Van Haastert et al., 2011), and non-cystic PVL now accounts for the majority of cerebral white matter injury observed in premature infants (Khwaja and Volpe, 2008).

Inder et al. showed that cerebral injury in premature infants typically manifests as diffuse white matter atrophy, ventriculomegaly, immature gyral development, and enlarged subarachnoid space (Inder et al., 2003). In addition, this group (Inder et al., 2005) have shown that white matter injury results in reductions in volumes of deep nuclear gray matter and cortical gray matter as well as disturbed white matter microstructure (Cheong et al., 2009).

In addition to these abnormalities, children born prematurely experience interruptions to their third trimester brain development, growth and gyration, and arrested development of the oligodendrocyte cell lineage which disrupts myelination and brain connectivity development (Back et al., 2001). Recent research has demonstrated that preterm infants are at greater risk of: (a) neurodevelopmental difficulties at 2 years (Woodward et al., 2006); (b) motor deficit at 4 years (Spittle et al., 2011); and (c) a range of functional outcomes at 7 years (Anderson et al., 2017). Moreover, preterm birth is a risk factor for lower IQ and poorer educational performance (Cheong et al., 2013). Doyle et al. have shown that biological influences on cerebral structure in early postnatal life have a greater influence on long term outcome than subsequent early childhood environmental factors (Doyle et al., 2015).

#### Intraventricular Hemorrhage

IVH is bleeding inside the lateral ventricles and occurs in up to 45% of extremely preterm-, and 30% of very preterm- infants (Mukerji et al., 2015). IVH is graded from mild (grade 1) to severe (grade 4), according to a grading system first described by Papile in 1978, and is based on the amount of bleeding into the ventricle, the presence of ventricular dilatation and involvement of the surrounding white matter (Papile et al., 1978). The presence of IVH poses a risk of subsequent post hemorrhagic ventricular dilatation, the risk of which increases from 5% with grade 1 IVH to over 80% with grade 4 (Murphy et al., 2002).

Risks of neurodisability and mortality increase with increasing grades of IVH in a similar fashion. Most studies report a higher likelihood of cerebral palsy with increasing severity of IVH (Mukerji et al., 2015). Beaino et al. reported significantly increased odds ratios of cerebral palsy at 5-years when comparing grade 4 to no IVH (Beaino et al., 2010). Contrastingly, cerebral palsy following grade 3 IVH is variably reported, but the likelihood is higher when post hemorrhagic ventricular dilatation occurs. Although bilateral IVH is common, parenchymal lesions are typically restricted to one side, and thus often lead to unilateral spastic cerebral palsy (De Vries et al., 2011).

## Cerebral Palsy

Brain injury detected in the newborn period is predictive of cerebral palsy (Linsell et al., 2016). White matter injury, chiefly PVL, and IVH are commonly reported in preterm infants later diagnosed with cerebral palsy (Kidokoro et al., 2014).

Cerebral palsy is an overarching term used to describe a group of disorders of movement and posture that occur due to damage to the developing brain during pregnancy, around the time of birth, or in the first 28 days of life. In many countries post-neonatal brain injury up until 2 years of age (such as stroke or near-drowning) is also included in the definition of cerebral palsy. Registry data in many parts of the world provides useful information about the cerebral palsy prevalence, type and topography related to gestational age. In Australia about 43% of people with cerebral palsy were born preterm, however this proportion is likely to be higher in the United States where a greater proportion of babies are born preterm (7% in Australia vs. 10% in the US) (Martin et al., 2018). Recently published register data shows that the incidence of cerebral palsy in preterm and term populations is starting to fall in high-income countries (1.4–2.1/1,000 live births) (Galea et al., 2018). Moreover, the percentage of children with moderate to severe disability is also declining (Galea et al., 2018).

It has been well established that the rate of cerebral palsy in preterm survivors is inversely related to gestational age (Himpens et al., 2008). A more recent review by Linsell et al. found that gestational age surprisingly did not predict cerebral palsy although included studies were 32 weeks or below (Linsell et al., 2016). It appears that after 27 weeks, rates of cerebral palsy drop off quite quickly. All subtypes of cerebral palsy can result from preterm brain injury although milder subtypes are most common.

### Types of Cerebral Palsy

Cerebral palsy can be classified by the type of movement disorder or the parts of the body that are affected. The most common type is spastic cerebral palsy accounting for about 85% of all cerebral palsy (ACPR Group, 2018). Dyskinetic cerebral palsy accounts for 7%, with ataxic and hypotonic cerebral palsy accounting for a combined further 7% (ACPR Group, 2018). Infants with cerebral palsy who are born preterm (<37 weeks') are most likely to have bilateral cerebral palsy, with a diplegic pattern more common than quadriplegia (44 vs. 19%), while unilateral cerebral palsy is diagnosed in about 28% of preterm babies with cerebral palsy (Towsley et al., 2011; Reid et al., 2014; ACPR Group, 2018). Where PVL is cystic and widespread, spastic quadriplegia is the most common outcome (Novak et al., 2017). In these more severe cases it is not just the white matter that is involved but also the other cerebral structures e.g., deep nuclear gray matter, leading to the description “encephalopathy of prematurity” (Volpe, 2009).

### Comorbidities

The risk for comorbidities with cerebral palsy is very high, and for the most part correlates with severity of the brain injury and physical disability. Common and disabling comorbidities and co-occurring functional impairments with cerebral palsy include: chronic pain (3 in 4); intellectual disability (1 in 2); hip displacement (1 in 3); epilepsy (1 in 4); behavior disorders (1 in 4); nonverbal (1 in 4); sleep disorders (1 in 5); vision impairment (1 in 10); and hearing impairment (1 in 25) (Novak et al., 2012).

### Early Detection of Cerebral Palsy

Although preterm infants are at increased risk of cerebral palsy (Spittle et al., 2018), a diagnosis of cerebral palsy has traditionally not been made until 12–24 months, unless a severe disability was clearly evident in the neonatal intensive care period (ACPR Group, 2018). An international clinical guideline, based upon systematic review evidence, indicates cerebral palsy can now be accurately identified in children as young as 12-weeks corrected age (Novak et al., 2017). Since no biomarkers yet exist for cerebral palsy, the guideline recommends using a combination of standardized assessment tools. The results of these tests should triangulate, with all the tests pointing in the direction of high-risk for cerebral palsy. If the test results are incongruent, the child may have another disability that is not cerebral palsy. The tools with the best psychometric properties for accurately predicting cerebral palsy by 3-months corrected age include: (1) Prechtl's General Movements Assessment (a video-based observation of the infant's spontaneous movement, scored for quality of movement) (98% sensitivity); (2) term-age equivalent MRI indicating damage to the motor areas of the brain (86–89% sensitivity); and (3) the Hammersmith Infant Neurological Examination (a scored neurological physical examination, with risk cut points determined from large sample studies) (90% sensitivity) (Novak et al., 2017). The results of these assessments should be carefully and compassionately communicated to the child's parents. Early diagnosis enables referral to early intervention to improve the child's outcomes, prevent secondary complications and protect parental mental health (Novak et al., 2017).

## Emergent Interventions

**Figure 1:**
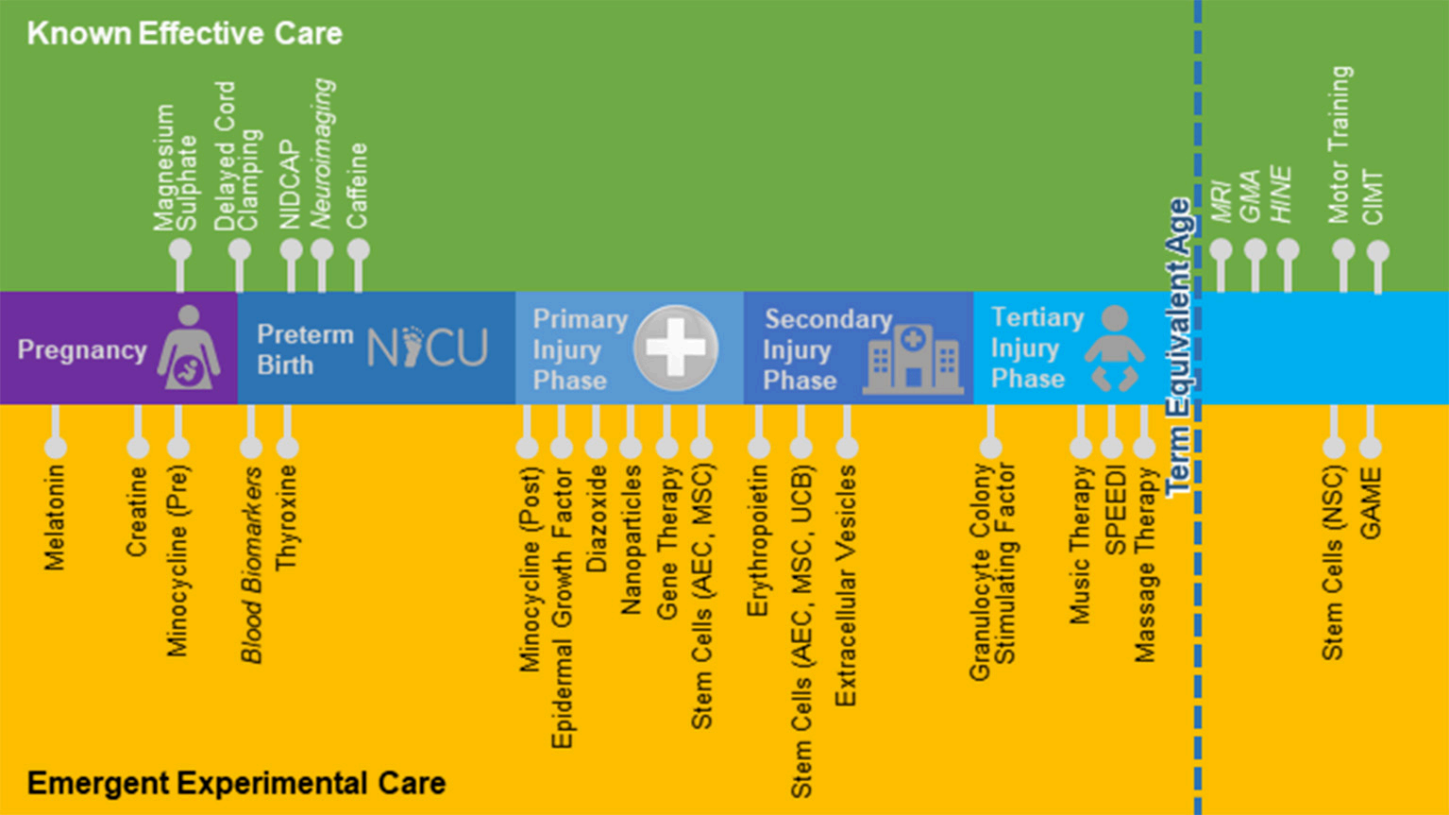
Known effective and emergent treatments and assessments. AEC, Amnion epithelial cells; CIMT, Constraint induced movement therapy; GAME, Goals Activity Motor Enrichment; GMA, General Movements Assessment; HINE, Hammersmith Infant Neurological Examination; MSC, Mesenchymal stem cell; MRI, Magnetic resonance imaging; NICU, Neonatal Intensive Care Unit; NIDCAP, Newborn Individualized Care and Assessment Program; NSC, Neural stem cell; SPEEDI, Supporting Play Exploration and Early Developmental Intervention; UCB, Umbilical cord blood.

There is a wide range of therapeutic interventions that have been proposed and are currently being tested to promote neurological repair in both infants and adults. Wherever possible, in this review, we present data generated from preclinical and clinical studies specifically utilizing preterm models and/or targeting premature populations. Where there is little published evidence, we have cast our net wider to include studies focusing on related neurological conditions such as adult stroke, traumatic brain injury and neurodegenerative conditions. Caution is warranted when interpreting the utility of interventions that are effective in adults back to the preterm infant, as the preterm baby is not simply a small adult, and their physiology often times contradicts the adult literature. Nevertheless, the results of some of these studies may be found to be relevant for preterm brain injury, and thus we see value in reviewing these studies, when there is little preterm-specific evidence available. In Table 1 we provide a summary of the interventions covered in this review, their mechanism of action, the current phase of research translation, route and timing of administration, plus, potential advantages and controversies to overcome through research. In addition, the timing of administration for known effective and emergent therapies for preterm infants is further illustrated in Figure 1.

**Table 1:** Potential therapeutic agents and research progress.

**Agent**	**Mechanism of** ** action**	**Sub-population of patients that might be treatment responsive**	**Research phase for preterm brain injury**				**Route of administration**	**Timing of administration**	**Advantages**	**Disadvantages/** **controversies**
			**Preclinical**	**Phase 1**	**Phase 2**	**Phase 3**				
Cell therapy|amnion epithelial cells (AEC) Cells obtained from the placenta	Anti-inflammatory and trophic TARGET = Motor, cognitive, behavioral and respiratory gains	BPD All preterm births as a universal neuro-protectant	✓ ✓	✓ ✗	✗ ✓	✗ ✗	IV IV	If 28-weeks GA and still requiring O2 at 36-weeks GA 3 doses in the first week of life	Proven safe for newborns in BPD Phase 1 trial (Lim et al., 2018) Easily obtained from placenta Morally uncomplicated source of cells Low immunogenicity Can be stored for later use Can be isolated and expanded to treat multiple patients Crosses	Unknown if it is better to administer AECs as a universal treatment for all preterm births at risk of disability, in the very acute phase OR whether to wait until a disability can be diagnosed and then offer a targeted AEC treatment later after injury
Cell therapy|mesenchymal stem cells (MSC) Multipotent adult stem cells, found in bone marrow and umbilical cord tissue and blood	Anti-inflammatory and trophic TARGET = Motor and cognitive gains	CP >6-months of age, of all sub-types and causal pathways IVH Perinatal stroke	✓ ✓ ✓ ✗	✓ ✓ ✗ ✗	✓ ✗ ✗ ✗		IV and intrathecal from autologous source, bone marrow derivedIntra- cerebroventricular injectionIntranasal and intra-arterial	Tested late in the tertiary phase of injury. Autologous MSC doses of 1–2 × 10^7^ Within 7 days of IVH On day 1 of injury	Proven safe in humans Can be obtained autologously from bone marrow or umbilical cord Available in off-the-shelf commercial formats for emergency and high/multi-dose administration Low immunogenicity Can be stored for later use Can be isolated and expanded Short survival following transfusion, lowering the risk of adverse events Some	Unknown if the cells might be more efficacious when transplanted stereotactically to increase the concentration of cells crossing the BBB Unknown if the cells might be more efficacious when transplanted in combination with other stem cell types with longer duration effects
									Bone marrow derived MSCs shown to be more effective than bone marrow mononuclear cells in a clinical trial for CP (Liu et al., 2017)	
Cell therapy|neural stem cells (NSC)Stem cells that make the 3 types of brain cells	Regenerative, anti-inflammatory and trophic TARGET = Cure or Motor and cognitive gains in tertiary phase injury	CP 1 year old and up, of all sub-types and causal pathways	✓	✓	✓	✗	Intracranial, stereotactically placed neurosurgically	Unknown, may be effective in the tertiary phase of injury as a cure	Regenerative capabilities Shown to be safe in human trials when transplanted with immunosuppression Can be stored for later use One donor's cells can be expanded to treat multiple patients	Must be obtained from embryos, fetal or iPSC sources, as it is unsafe for adults to donate Neurosurgical
Cell therapy|umbilical cord blood (UCB) Umbilical cord blood rich in an array cell types including stem cell populations	Anti-inflammatory and trophic TARGET = Motor and cognitive gains in late tertiary phase treatment; disability-free survival in primary and secondary phase treatment	CP 1 year old and up, of all sub-types and causal pathways HI injury All preterm births as a universal neuro-protectant	✓ ✓ ✓	✓ ✓ ✗	✓ ✓ ✗	✗ ✗ ✗	IV (Autologous and allogeneic)IV (autologous)IV (allogeneic)	Tested late in the tertiary phase of injury. Autologous UCB doses >2 × 10^7^/kg produced better results (Sun et al., 2017) After 6 h of injury and initiation of hypothermia. Multiple doses in the first week of life Unknown	Proven safe in humans Easily obtained from umbilical cords Morally uncomplicated source of cells Source can be autologous or allogeneic and can be HLA matched Can be stored for later use Theoretically	Difficult to collect and reinfuse autologous cords in an emergency birth with HI injury Unknown risks of autologous reinfusion for genetic and/or infection causal pathways to CP, i.e., could the injury be worsened? Preterm infants have small low volume cords with a different cell make-up to term cords, and therefore autologous infusions may have less therapeutic value
										Efficacy appears higher with higher HLA matching, meaning large, diverse cord banks are needed Need for off-the-shelf expanded products for emergency and/or high-dose/repeat treatments Unknown GVHD risk from combining allogeneic cords to deliver high/multi-dose treatments Unknown effect of delayed cord clamping on cord volumes and efficacy
Extracellular vesicles (EVs) Membrane fragments released from cells (exosomes and microvesicles) with neuroprotective effects	Anti- inflammatory, restoring myelination and cell microstructure TARGET = Cognitive gains	Preterm stroke HI	✓	✗	✗	✗	IV	Unknown, but presumably in the primary and secondary phases of injury	Safer alternative to stem cells Can be stored for later use Collected in large volumes Administered off-the-shelf Low immunogenicity Can transport cargo Crosses	Autologous preterm derived EVs may lack sufficient aerobic potential to be therapeutic and therefore allogeneic term equivalent sources may be required
Erythropoietin (EPO) Natural red blood cell hormone with neuroprotective effects. Also manufactured synthetically enabling dose titration	Anti-inflammatory, anti-excitotoxic, anti-oxidant, trophic, plus enhanced neurogenesis and angiogenesis	IVH Hemorrhagic parenchymal infarction HI	✓	✓	✓	✓	IV	3 doses of 1,000 U/kg of EPO in the first week of life, during the secondary and tertiary phases of injury (Wu et al., 2012)	Proven safe in neonates Administered off-the-shelf Can be administered in the tertiary phase of injury	Cost Refrigeration requirements Lack
	TARGET = Cognitive (and motor) gains									Some infants will be discharged from NICU before all 3-doses can be administered
MelatoninNatural hormone that regulates circadian rhythm. Also manufactured synthetically enabling dose titration	Anti-inflammatory and anti-apoptotic TARGET = Motor and cognitive gains	IUGR HI injury	✓ ✓	✓ ✓	✓ ✗	✗ ✗	Oral (Maternal)Enteral	Antenatal 3x daily 10 mg, from baseline until birth 0.5–3-5 mg/kg dose escalation in first 6 h of life	Administered off-the-shelf Low cost Proven	Large sample sizes required to run Phase 3 public health trials, powered to detect a protective benefit
CreatineNatural diet compound that builds muscle, with neuroprotective effects	Anti-excitotoxic and anti-apoptotic TARGET = Disability-free survival from HI injury	Mothers with pre-eclampsia, cervical incompetence, placental abruption, placental previa, IUGR TBI as proof of concept for HI injury	✓ ✓	✗ ✓	✗ ✓	✗ ✗	Oral diet supplementation (Maternal)Oral	Antenatal, especially during the 3rd trimester of pregnancy 0.4 g/kg daily for 6-months duration	Proven safe in humans Straightforward bioavailability Administered off-the-shelf Low cost Simple and feasible intervention for patients to adhere to Manufactured synthetically enabling dose titration May have the added benefit of reducing labor pains	Large sample sizes required to run Phase 3 public health trials, powered to detect a protective benefit
Granulocyte-colony stimulating factor (G-CSF) hematopoietic growth factor	Stimulate neural stem and progenitor cell production TARGET = Motor gains	CP 2-10 years old, all sub-types and causal pathways	✓	✓	✓	✗	IV	10 μg/kg for 5 days. Should be used >60 h after injury	Appears	Unclear
ThyroxineThyroid hormone	Normalization of thyroid levels as a neuroprotectant TARGET = Disability free survival	Hypothyroxinemia IVH	✓	✓	✓	✓	IV	Birth to 42-days	Proposed reduced mortality Proposed	Clinical trials indicated improved long-term neurodevelopmental outcomes but these results are not supported in a meta-analysis
MinocyclineBroad-spectrum antibiotic	Inhibition of microglial activation TARGET = Motor and cognitive gains	HI	✓	✗	✗	✗	IV	Before and after injury	Promising preclinical data indicating a reduction in white matter damage	Conflicting results in human studies of other adult degenerative neurological disorders, with some studies showing harm Timing
Epidermal growth factor (EGF)Growth factor	Regulation of NSC migration, proliferation and oligodendrocyte differentiation to increase myelination TARGET = Motor gains	Focal demyelination HI injury IVH	✓	✗	✗	✗	Overexpression of EGF receptor in oligodendrocytes Intranasal Intra-cerebroventricular injection	Before and after injury Immediately after injury 3 doses at 24, 72 and 120 h post-IVH	Preliminary preclinical data indicating increased myelination and functional recovery	Cost Can induce neurotoxicity Might
DiazoxideVasodilator	Prevention of hypoglycemia TARGET = Motor and cognitive gains	HI	✓	✗	✗	✗	Intraperitoneal	Daily during induction of HI injury	Well established safety profile (from other indications) Rapid acting Inhibits	Does not cross the BBB but affects circulation May cause hypotension Fluid retention
Nanoparticles Microscopic particles including dendrimers (branched molecules that can be tailored to store and transport materials)	Drug or gene transporter TARGET = Motor gains; reduction in rates of preterm birth	HI injury Infection and PVL	✓	✗	✗	✗	IV	Unknown, but presumably in the primary, secondary and tertiary phases of injury when inflammation is present	Can transport cargo, including anti-inflammatory agents Crosses the BBB Targets abnormal microglia and astrocyte activity Low immunogenicity	High manufacturing costs Potential toxicity
Gene TherapyDelivery of DNA as a drug	Anti-excitotoxic to excessive glutamate via gene delivery of BDNF TARGET = Motor gains via protection of white matter volume loss	PVL	✓	✗	✗	✗	Intracerebellar	Research indicates in the primary and secondary phases of injury (Gressens et al., 1997)	Unknown	Prolonged administration required because precise timing of the insult is usually unknown Neurosurgical administration required
Parent attachment training Teaching parents to read and respond sensitively to their infant's cues	Experience dependent plasticity	IVH PVL	✓	✓	✓	✗	Parent Education Manual guidance	1 h weekly	Lasting benefits to cognition	Heterogeneous, under powered samples Limited effect on motor skills (Nelson et al., 2001; Ohgi et al., 2004)
NIDCAP A low stress and low stimuli NICU environment that mimics the uterine environment	Experience dependent plasticity	IVH PVL Stroke HIE	✓	✓	✓	✓	Adapted environment e.g., low lighting and noise	24/7	Possible short term protection of brain structure	No long term benefits conferred (Ohlsson and Jacobs, 2013)
SPEEDI Parent coaching and environmental enrichment	Experience dependent plasticity Parent Education	IVH WMI HIE Hydrocephalus	✓	✓	✓	✗	Adapted environment e.g., promoting child active learning	21 days + 12 weeks of daily practice	Improved problem solving (Dusing et al., 2018)	Heterogeneous, under powered samples
GAME Task-specific motor training, parent coaching and environmental enrichment	Experience dependent plasticity Parent education	IVH PVL Stroke HIE	✓	✓	✓	✓	Adapted environment Task practice	Daily 45 min of practice by the child (Morgan et al., 2016b)	Improved motor skills Improved cognitive skills	Co-occurring vision impairment may lower the rate of gains

*✓ = Yes; ✗ = No; AEC, Amnion epithelial cells; BBB, Blood brain barrier; BDNF, Brain-Derived Neurotrophic Factor; BPD, Bronchopulmonary dysplasia; CP, Cerebral palsy; CIMT, Constraint induced movement therapy; DNA, Deoxyribonucleic acid; EGF, Epidermal growth factor; EPO, Erythropoietin; EVs, Extracellular vesicles; g, grams; GA, Gestational age; GAME, Goals Activity Motor Enrichment; GVHD, Graft vs. host disease; HI, Hypoxic ischemic; HIE, Hypoxic ischemic encephalopathy; HLA, Human Leukocyte Antigens; iPSC, Induced pluripotent stem cell; IUGR, Intrauterine growth restriction; IV, Intravenous; IVH, Intraventricular hemorrhage; kg, kilograms; μg, micrograms; mg, milligrams; MSC, Mesenchymal stem cell; MRI, Magnetic resonance imaging; NICU, Neonatal Intensive Care Unit; NIDCAP, Newborn Individualized Care and Assessment Program; NSC, Neural stem cell; O2, Oxygen; PVL, Periventricular leukomalacia; SPEEDI, Supporting Play Exploration and Early Developmental Intervention; TBI, Traumatic brain injury; U, Units; UCB, Umbilical cord blood; WMI, White Matter Injury*.

### Biological and Pharmacological Therapies

#### Cell-Based Therapy

Cell therapies offer great promise for treating neurological diseases and are emerging as a new paradigm in human medicine (Trounson and Dewitt, 2016). The mechanisms by which candidate cell therapies might work for brain injury include (1) anti-inflammatory mechanisms: attenuation of the inflammatory immune response to brain injury via a reduction in the release of excitotoxins, cytotoxins, and reactive oxygen species; (2) trophic mechanisms: to promote cell survival via release of neurotrophic factors to induce endogenous cell migration, proliferation and differentiation and/or promote angiogenesis; and (3) regenerative mechanisms: replacement of damaged brain tissue by engraftment, proliferation and differentiation of transplanted cells (reviewed in Novak et al., 2016). Different types of cells have been proposed, including: amnion epithelial cells (AECs); mesenchymal stromal/stem cells (MSCs); umbilical cord blood (UCB), and neural progenitor/neural stem cells (NSCs) (Novak et al., 2016), each with different advantages and disadvantages that must be considered when contemplating therapeutic application. Substantial preclinical evidence now exists to support the conduct of human trials evaluating efficacy of various cell therapies for perinatal brain injury (reviewed by Fleiss et al., 2014).

There are ethical complexities and controversies to be considered when conducting clinical trials of stem cells in newborns with perinatal brain injury. First, these children have a lot to gain but also a lot to lose from an adverse event. Thus, human ethics committees often favor “first in human” studies to be conducted in adult populations where the patient can consent, rather than consent being obtained by proxy from a parent. Hence there have been no clinical trials in preterm infants where the cells have been delivered in the acute newborn period. Second, not all preterm babies have a disability. Accurate determination of which patients will have a normal outcome and which patients will have a disability can take months, and even years for subtle literacy problems in reading and writing to be apparent. Therefore, the dilemma exists about timing of stem cell treatment for preterm infants. Should universal treatments be applied to all preterm patients in the newborn period given that the risks of disability are high, and the newborn period is the optimal therapeutic window in terms of an effect in the acute phase? Or should we wait and provide targeted therapies once the children with definite long-term disability can be identified, all the while lowering the potential treatment effect size from late intervention in the chronic phase of brain injury? Third, the mechanism of action should match the clinical indication. Cells that have anti-inflammatory and trophic properties (i.e., AECs, MSCs, and UCB) will theoretically will be of most benefit during the acute period of neuroinflammation, if neuro-repair is sought. If administered in the chronic phase, smaller gains may still be possible via trophic effects and the emerging evidence that inflammation persists into the tertiary phase of injury (Fleiss and Gressens, 2012). Contrastingly, cells that have regenerative capacity (i.e., NSCs) are a better therapeutic target for chronic stage injury. Fourth the origin of the cells can be controversial for some. AEC, MSC, and UCB cells can be obtained using morally uncomplicated methods: AECs are obtained from placental tissue under maternal consent, UBC from cords with maternal consent, and MSCs are available in off-the-shelf commercial formats or can be obtained autologously from bone marrow harvesting with patient consent. NSCs cannot be safely obtained from adult donors and therefore need to be obtained with consent from embryonic or fetal sources, or grown autologously from induced pluripotent cells.

Given the challenges of designing stem cell trials for preterm infants, there are limited published studies. However, Rudnicki et al. assessed the safety and feasibility of autologous UCB for extremely preterm (<32 weeks') neonates who developed anemia due to prematurity (Rudnicki et al., 2015). In this trial, infants received an intravenous transfusion of 15 mL/kg of body weight of either autologous UCB (*n* = 5) or allogeneic red blood cells (*n* = 9; control group), administered on average 3.2 and 7.8 days after birth for UCB and red blood cells, respectively. Results from this small study group indicated that autologous UCB transplantation in preterm newborns was generally safe and well tolerated. Recently published, Won Soon Park's team at Samsung Medical Center in South Korea conducted a Phase 1 dose-escalation study of Pneumostem® transplantation in preterm infants with severe IVH (grade 3–4) (NCT02274428) (Ahn et al., 2018). Pneumostem® is a human allogeneic UCB-derived MSC product. Eligible infants were born at 23–34 weeks' gestation, and received cells (5 × 10^6^ cells/kg or 1 × 10^7^ cells/kg) within 7 days of IVH diagnosis. Study results indicated that Pneumostem® transplantation by intracerebroventricular injection was safe; cell transplantation at both doses was not associated with any serious adverse events or dose-limiting toxicity, and there were no cases of mortality in their cohort of 9 infants (Ahn et al., 2018). This is being followed up by a currently-recruiting Phase 2a study to evaluate the efficacy and safety of intraventricular administration of Pneumostem® for treatment of IVH (grade 3–4) in 22 high-risk premature infants (NCT02890953). Participants will be randomized to receive either Pneumostem® or saline control within 28 postnatal days. Outcomes include death, ventricular shunt operation, and ventricular dilatation.

There are now multiple clinical trials underway examining the safety and efficacy of cell therapies (chiefly MSCs and AECs) for other morbidities associated with prematurity, in particular bronchopulmonary dysplasia. It is feasible that these therapies may provide non-specific neuro-protective benefit through reduction of systemic inflammation. These trials should therefore be designed (e.g., length of follow up), and sufficiently powered, to capture any potential neurological benefits of these therapies. As an example, the long-term safety and efficacy follow-up study of Pneumostem® (NCT02023788) in patients who completed the previously mentioned Phase I study (NCT02274428) includes neurological development test outcomes at 5 years (corrected age) comprising the K-ASQ (Korean Ages and Stages Questionnaires) and Bayley Scales of Infant and Toddler Development (Bayley).

Although not specifically targeting preterm infants, the following clinical trials warrant mention. First is the open label trial of 23 term-born neonates (≥35 weeks') with hypoxic ischemic encephalopathy (HIE) who received non-cryopreserved autologous volume- and red blood cell-reduced UCB intravenously (Cotten et al., 2014). Infants received up to four infusions of 1–5 × 10^7^ cells/kg, with the first dose as soon as possible after birth, and at 24, 48, and 72 postnatal hours. The authors found that whilst autologous UCB for infants with HIE is feasible, UCB collection at delivery for all “obstetric emergencies” was a challenge that initially resulted in extremely low recruitment rates; they originally intended to recruit 52 HIE infants, however only enrolled 23. They go on to describe how this challenge was overcome by multidisciplinary collaboration. Nevertheless, timely collection of autologous UCB may be a potential barrier for this therapy in certain settings. There are now multiple currently-recruiting Phase 1 and 2 studies of autologous UCB for HIE listed on clinical trial registries. These include the follow-on study (NCT02612155) at Duke University, as well as other studies in the United States (NCT02434965), France (NCT02881970), and China (NCT02551003, NCT03352310). Finally, for a different indication, there is the phase 1/2 open-label study of intranasally administered bone marrow-derived allogeneic MSCs (5 × 10^7^ cells) in term neonates (≥36 weeks') with perinatal arterial stroke (PAIS) (NCT03356821). Referred to as the PASSIoN trial, this study aims to recruit 10 participants for cell administration as soon as possible after confirmation of PAIS (maximum within the first week). PAIS is an important perinatal cause of long-lasting neurodevelopmental problems.

Due to the limited number of published clinical trials of stem cells for perinatal/preterm brain injury, it is not surprising that no systematic reviews have yet been published. However, three reviews with meta-analyses have been conducted analyzing stem cell clinical trials for other indications including two for adult stroke and one for children with cerebral palsy. Interestingly, meta-analysis of the effects of MSCs for ischemic stroke, which included seven studies, indicated no significant difference between stem cell and cell-free treatments (Wang et al., 2016). This was in contrast to the analysis of Chen et al., which revealed stem cell transplantation for patients with ischemic stroke can significantly improve neurological deficits, motor function, daily life quality and functional independence (Chen et al., 2016). This analysis included 18 studies, the majority of which used MSCs (*n* = 10) however neural stem cells (*n* = 4), bone marrow mononuclear cells (*n* = 2), peripheral blood cells (*n* = 1), and umbilical cord MSCs (*n* = 1) were also used (Chen et al., 2016). It is therefore possible that cell types other than MSCs contributed to the larger effect size observed. For children with cerebral palsy, stem cells appeared to induce short-term improvements in gross motor skills, despite the acknowledged limitation of heterogeneous data e.g., cell type, participant age range and cerebral palsy sub-type (Novak et al., 2016). Collectively, these papers conclude that further research, in particular randomized controlled trials (RCTs), using rigorous methodologies, is warranted to determine the optimal stem cell treatments for neurological conditions.

#### Extracellular Vesicles (EVs)

MSCs, and likely other types of cells, exert their neuro-protective/reparative effects via secretion of extracellular vesicles (EVs), namely exosomes and microvesicles (reviewed by Willis et al., 2017). EVs are small membrane-bound particles with a size of 70–1,000 nm that contain an array of bioactive “cargo” which includes DNA, messenger RNA, microRNA, proteins, and lipids (Willis et al., 2017). EVs offer an attractive option as a novel, cell-free alternative therapy for the treatment of perinatal brain injury, since their use eliminates some of the largest perceived risks of cell therapy: tumorigenic potential of administered live cells, as well as immunologic reactions and/or rejection of transplanted cells. Another benefit of EVs is that they can be easily collected in large quantities and stored for future use (Willis et al., 2017), providing “off-the-shelf” capability for clinical emergencies.

The efficacy of EVs for perinatal brain injury is still being explored in preclinical studies, including a rodent model of inflammation-induced preterm birth with resultant brain damage (Drommelschmidt et al., 2017), and a preterm sheep model of hypoxic-ischemic (HI) brain injury (Ophelders et al., 2016). In rats, MSC-EVs ameliorated inflammation-induced cellular damage, restoring myelination deficits and abnormal microstructure, indicated by reduced fractional anisotropy measured using diffusion tensor MRI, resulting in improved long-term cognitive function assessed using the spatial probe test as an indicator for adaptive memory function (Drommelschmidt et al., 2017). Similarly in sheep, MSC-EVs partially rescued brain function following HI-induced injury, and appeared to mildly protect against hypomyelination (Ophelders et al., 2016). Additionally, there are numerous published studies demonstrating the utility of EVs for adult brain injury, in particular traumatic brain injury (TBI) (Zhang et al., 2015, 2017b; Kim et al., 2016; Li et al., 2017; Williams et al., 2019), and stroke (Otero-Ortega et al., 2018). Together, these studies highlight the potential of EVs for the treatment of brain injury, and more research into their use for neurological conditions is merited. Lessons learned from EV studies using these models may be beneficial for preterm indications.

An important consideration for EV therapy is the source of the EVs and the timing of retrieval. EVs can be derived from different cell types. It seems logical to favor autologous products because, as for cell-based therapies, they are considered safer due to the eliminated risk of immune rejection of the cell product or development of graft-versus-host disease, or transmission of infectious diseases from donor to recipient. Intriguingly, a 2016 study presents evidence that autologous EVs for preterm infants might not be best choice (Panfoli et al., 2016). In this study, exosomes derived from umbilical cord-MSCs of preterm babies (28–30 weeks' gestation) had reduced aerobic potential compared to those derived from term babies (≥37 weeks') (Panfoli et al., 2016). Reduced aerobic potential might affect their ability to rescue the bioenergetics of damaged tissues (i.e., by restoring ATP and NADH levels). Thus, preterm exosomes may have a diminished capacity to provide therapeutic benefit, which may have design implications for EV-therapies in preterm infants.

Interestingly, EVs may also be utilized to deliver gene therapy to target cells. In a series of *in vitro* experiments, Lee et al., used MSCs genetically modified to express fluorescently labeled microRNA-124 to demonstrate that MSCs can deliver microRNA to neural cells via the secretion of exosomes (Lee et al., 2014). MicroRNA-124 was selected because it plays a role in neurogenesis, indeed miRNA-124 is the most abundant miRNA in the brain, which suppresses cell proliferation, promotes neuronal differentiation and neurite outgrowth, and represses astrocytic differentiation (reviewed in Sun et al., 2015). Consistent with this, delivery of microRNA-124 induced neuronal differentiation in targeted neural progenitor cells. This concept has now progressed to clinical trial: a Phase 1 trial will examine the safety and efficacy of allogeneic MSC-derived exosomes “enriched” with microRNA-124 in five adults (40–80 years) with acute ischemic stroke (NCT03384433). MicroRNA-124 enrichment will be achieved via transfection of MSCs. Outcome measures include the incidence of treatment-emergent adverse events and the Modified Rankin Scale to measure change in disability or dependence in daily activities. Other developments in the EV clinical trial space include published studies of chronic kidney disease (Nassar et al., 2016) and graft-vs.-host disease (Kordelas et al., 2014), as well as the currently recruiting study for healing of large and refractory macular holes using MSC-derived exosomes (NCT03437759).

Given the potential advantages of EVs (Hall et al., 2016), this raises an ethical controversy of whether researchers should be progressing predominantly EV- rather than cell-based- therapies. Studies such as that published by Doeppner et al., which compared the efficacy of human bone marrow-derived MSCs with EVs obtained from the same cells in a rodent model of stroke (Doeppner et al., 2015), are highly valuable. This study showed that EVs were equally effective as cells in promoting neurological recovery and brain remodeling following transient focal cerebral ischemia. More such head-to-head studies are warranted to confirm the utility of EVs for a range of indications. The efficacy of EVs for neuro-regeneration/repair is expected to be limited to situations that involve anti-inflammatory and/or trophic signaling mechanisms; it is unlikely that EVs would be useful when cell engraftment is desired, for example in neural stem cell replacement therapy.

#### Erythropoietin (EPO)

Originally identified for its role in erythropoiesis, erythropoietin (EPO) was later shown to be neuroprotective in a variety of animal models of neurological conditions including stroke, TBI and multiple sclerosis (MS) (Siren et al., 2009). Unsurprisingly, EPO garnered significant interest as a potential neuroprotectant for preterm infants, and numerous preclinical- and clinical-studies have since been completed. Three recent systematic reviews with meta-analyses of EPO for reducing neurodevelopmental disability in preterm infants have been published. These studies comprised: four RCTs with 297 infants (Zhang et al., 2014), two RCTs and three quasi-RCTs with 233 infants (Wang et al., 2015), and four RCTs with 1,133 infants (Fischer et al., 2017), in the meta-analyses, respectively. Analyses were conducted on mostly non-overlapping trials: seven original studies in total, and all three concluded that EPO treatment improved cognitive outcome in preterm infants. Important to note is that no significant effect was observed for other neurodevelopmental outcomes including cerebral palsy, visual impairment, severe hearing deficit, and necrotizing enterocolitis.

New clinical trials of EPO for preterm infants continue to be launched. A currently recruiting, multicenter, randomized, placebo-controlled study plans to recruit 312 infants (<32 weeks' gestation, ≤1,500 grams) for repeated administration of high-dose EPO within 48 h of birth (NCT02550054). The primary outcomes are neurodevelopmental function at 18 months measured using the Bayley and GMFM-88. Similarly, “EpoRepair” (NCT02076373) is an RCT of repetitive high-dose EPO in 120 preterm infants (23–31 weeks'). Contrastingly however, recruited infants will be those with a diagnosed brain injury (IVH and/or hemorrhagic parenchymal infarction), and neurodevelopmental follow-up using a composite intelligence quotient will be conducted at the longer time-point of 5 years of age (Ruegger et al., 2015). Moreover, the active, though not recruiting Phase 3 Preterm Erythropoietin Neuroprotection “PENUT” Trial (NCT01378273) has enrolled 941 preterm infants specifically in the lower age range of 24–27 weeks' gestation. Infants in this study will likewise receive repeated high-dose EPO initiated soon after birth. The primary endpoint is 24–26 months corrected age, at which neurodevelopmental outcome will be measured using the Bayley (Juul et al., 2015). The selection of extremely low gestational age neonates for the PENUT Trial is significant since in a subgroup analysis reported by Fischer et al., no significant benefit of EPO was seen on cognitive outcome in infants <28 weeks' (Fischer et al., 2017). Whilst the authors acknowledged that the small sample size used for the analysis was a limitation, the results of the PENUT Trial should definitively address the efficacy of EPO for neuroprotection in extremely preterm infants. Of particular note, whilst the EPO regimens of the above listed trials differ, the effect of EPO on cognitive outcome appears to be robust enough to withstand variations in both timing and dose (Fischer et al., 2017). Thus, hopefully the results of these studies will provide conclusive evidence to support change in the clinical practice guidelines for the care of preterm infants.

#### Melatonin

Melatonin (N-acetyl-5-methoxytryptamin) is a hormone produced by the pineal gland that is responsible for regulating circadian rhythms via activation of specific melatonin receptors. Melatonin also acts as an antioxidant and has demonstrated anti-inflammatory and anti-apoptotic effects (Welin et al., 2007). It is these properties that make melatonin a promising candidate for the treatment of perinatal brain injury in preterm neonates. Accordingly, there is now substantial evidence from animal studies supporting a neuroprotective role of melatonin administered either antenatally, or postnatally after the injurious event (reviewed in Biran et al., 2014; Wilkinson et al., 2016). For example, in a murine model of excitotoxic periventricular white matter injury, which mimics human PVL, melatonin given immediately following intracerebral injection of the glutamatergic analog ibotenate promoted secondary lesion repair, effectively reducing the size of white matter cysts (Husson et al., 2002). Similarly, in a rat model of acute neonatal hemorrhagic brain injury commonly observed in very low birth weight preterm infants, systemic administration of melatonin 1 h after injury normalized brain atrophy and led to improved cognitive and sensorimotor function (Lekic et al., 2011). Moreover, Watanabe et al., demonstrated that antenatal administration of melatonin reduced *in utero* ischemia/reperfusion oxidative brain injury in neonatal rats. Rat pups born to mothers who received melatonin exhibited reduced markers of oxidative stress, had numbers of intact mitochondria comparable to control animals, and had reduced brain damage quantified by protection of hippocampal pyramidal neurons from ischemia/reperfusion-induced degeneration (Watanabe et al., 2012). Likewise, maternal antenatal melatonin reduced brain injury in an ovine model of fetal growth restriction (Miller et al., 2014). In this model, fetal growth restriction was induced surgically by single umbilical artery ligation at 0.7 gestation after which melatonin was administered for the remainder of the pregnancy. Antenatal melatonin rescued oxidative stress, white matter hypomyelination and axonal damage leading to significant functional improvements in neonatal lamb behaviors, in particular suckling (Miller et al., 2014). Collectively, these studies demonstrate the broad utility of melatonin in improving outcomes following perinatal injury from a variety of etiologies. In addition, there is encouraging data on the neuroprotective efficacy of melatonin in a range of related adult conditions including stroke (Watson et al., 2016), TBI (Barlow et al., 2018), and neurodegenerative diseases (Wongprayoon and Govitrapong, 2017).

The results of these pivotal animal studies have led to pilot clinical trials of either antenatal or postnatal melatonin for neuroprotection of preterm or growth restricted infants. Though few trials have been published to date, a single-arm, open-label study of antenatal oral melatonin administration to 16 women with a growth restricted fetus (diagnosed <34 weeks gestation) was completed in November 2014 (NCT01695070) (Alers et al., 2013). This study is being followed by the 2017-registered “Protect Me Trial” which aims to recruit 292 mothers with a growth restricted fetus for a Phase 2 RCT of daily antenatal melatonin supplementation for improving early childhood neurodevelopmental outcomes at 2-years (ACTRN12617001515381). Trial participants will be stratified according to gestational age (<28 weeks' and 28 to <32 weeks'), and recruitment is anticipated to start in late-2018. For postnatally administered melatonin, a pharmacokinetic study was conducted in which 18 preterm infants (<31 weeks') were administered melatonin intravenously at varying concentrations for up to 6 h (NCT00649961). The objective was to determine the dose required to achieve melatonin blood levels in the preterm infant similar to that of the mother, to guide potential future therapeutic trials. It was found that peak adult melatonin concentration could be achieved by a relatively short (2 h) infusion of low dose (0.1 μg/kg/h) melatonin in preterm infants (Merchant et al., 2013). Using this dosing regime, a Phase 2 RCT (The Mint Study) was conducted to evaluate the neuroprotective effect of melatonin in 58 preterm infants (30 received melatonin and 28 received placebo) (Merchant et al., 2014). No between group difference was shown for the primary outcome, which was a 5% difference in the fractional anisotropy in the white matter measured using MRI at term equivalent age. Similar to the Mint Study, “PREMELIP” aimed to assess the neuroprotective effect of melatonin administered in the immediate prepartum period in very preterm infants (<28 weeks') (NCT02395783). Also designed to measure white matter injury at term equivalent age using MRI as the primary outcome, this study was terminated in 2018 after recruiting 14 participants. No reason was provided by study investigators. Melatonin is also being studied in *term*-born neonatal populations (Aly et al., 2015), however this is beyond the scope of this review.

#### Creatine

Creatine is a natural compound that is synthesized in the liver, kidney and pancreas, and is also obtained through diet. Creatine's main role is in cellular energy homeostasis; creatine participates in a reversible reaction with creatine kinase to continuously and efficiently replenish ATP (the energy currency of the cell) from ADP, to meet energy demands (Wallimann et al., 1992). There is evidence to suggest that creatine is neuroprotective for the developing perinatal brain (Dickinson et al., 2014b). Specifically, offspring born to pregnant spiny mice fed a creatine-supplemented diet had a significantly increased capacity to survive a hypoxic birth event, and these pups showed improved postnatal weight gain (Ireland et al., 2008). In a follow-up mechanistic study, maternal creatine supplementation protected pups from hypoxia-induced brain lipid peroxidation and apoptosis, hypothesized to be mediated via preservation of mitochondrial function (Ireland et al., 2011). However, no clinical trials of antenatal creatine supplementation to protect against perinatal brain injury have yet been conducted (Dickinson et al., 2014a).

Complexities exist around the practicalities of antenatal creatine administration for neuroprotection of preterm infants. For example, should creatine be administered as a prophylactic to all pregnant women on the off chance that their babies experience birth asphyxia and/or prematurity? Alternatively, the utility of postnatal creatine for neuroprotection of preterm infants who do not experience birth asphyxia is unclear. Creatine has been pilot tested in clinical trial for children with TBI, with daily administration from the day of injury to 6 months post-injury. The results suggested improved memory and functional skills, with reduced intensive care stay (Sakellaris et al., 2006). Thus, creatine could potentially be given postnatally to newborns with encephalopathic brain injury at high-risk of cerebral palsy. Notably, creatinine (the breakdown product of creatine) is excreted by the kidneys, which are known to have reduced function in premature infants (Stritzke et al., 2017). Thus, the effects of administering potentially high therapeutic doses of creatine to preterm infants with reduced kidney function warrants careful consideration and thorough investigation. Creatine is also being studied in adult neurodegenerative diseases (Pastula et al., 2012; Xiao et al., 2014; Bender and Klopstock, 2016), however these are beyond the scope of this review.

#### Granulocyte-Colony Stimulating Factor (G-CSF)

Granulocyte-colony stimulating factor (G-CSF) is a hematopoietic growth factor that is used clinically in cancer patients diagnosed with neutropenia following chemotherapy, due to its ability to stimulate hematopoietic stem cell mobilization and neutrophil differentiation. G-CSF also exhibits non-hematopoietic neurotrophic functions, with evidence from experimental studies indicating that G-CSF is neuroprotective in stroke, TBI, and neurodegenerative diseases (reviewed by Solaroglu et al., 2007). Despite this, results of studies examining the effect of G-CSF in perinatal brain injury were initially controversial. While two studies of HI injury in neonatal rats showed that G-CSF inhibited neuronal apoptosis to attenuate brain damage (Yata et al., 2007; Kim et al., 2008), Keller et al., found that G-CSF actually increased cortical and white matter lesions in a newborn mouse model of excitotoxic brain injury (Keller et al., 2006). Similarly, in a follow-up study, acute administration of G-CSF led to increased hippocampal brain damage in a murine model of neonatal HI (Schlager et al., 2011). This disparity was explained 5 years later, when it was revealed that the neuroprotective effects of G-CSF are contingent on the timing of administration following the insult (Neubauer et al., 2016). Delayed administration of G-CSF (in combination with stem cell factor or flt3-ligand) at 60 h post-injury, compared to 1 h used previously, conveyed significant neuroprotection against excitotoxic insult (Neubauer et al., 2016). This was consistent with other studies showing G-CSF reduced white matter injury in a sheep model of preterm brain injury (Jellema et al., 2013), and attenuated neuroinflammation and stabilized the blood brain barrier (BBB) following HI (Li et al., 2015). However, it remains unclear why Schlager et al., failed to find any long-term neuroprotective effects of G-CSF after neonatal HI (Schlager et al., 2011).

Thus far, the only clinical trials assessing the neuroreparative potential of G-CSF in pediatric patients has been in children with cerebral palsy (NCT02983708, NCT02866331). In the first study (NCT02983708), G-CSF was given to 57 children with non-severe cerebral palsy for 5 days to mobilize peripheral blood mononuclear cells. These were then collected and re-infused at either 1- or 7-months post-cell collection, in a double-blind, cross-over study (Rah et al., 2017). Neurodevelopmental improvement, above what would normally be expected, was seen in 42% of patients in response to G-CSF treatment. Interestingly, larger improvements in test scores were observed in patients who received delayed cell re-infusion at 7 months, although this was in contrast to the results of the MRI analysis. The authors concluded that further studies are needed to delineate the effect of repeated G-CSF either alone, or in combination with reinfusion of G-CSF-stimulated peripheral blood cells. The second study, a subsequent trial from the same lead investigator, is currently recruiting (NCT02866331). In this study, the efficacy of autologous UCB mononuclear cells and/or repeated G-CSF administration for children with cerebral palsy will be assessed. In addition to these limited pediatric studies, numerous clinical trials of G-CSF for adult stroke have been completed. Meta-analyses of clinical trial data however paint a less-than-convincing picture, with conflicting conclusions (Fan et al., 2015; England et al., 2016; Huang et al., 2017). There has also been substantial interest in G-CSF for ALS, however early phase clinical trials have failed to show efficacy (Cashman et al., 2008; Nefussy et al., 2010; Chio et al., 2011; Duning et al., 2011). This serves as a timely reminder that compelling preclinical results, like creatine for neurodegenerative conditions, does not always translate to clinical benefit.

#### Thyroid Hormone (Thyroxine)

The thyroid hormones (thyroxine and triiodothyronine) are essential for brain development during fetal and postnatal life (reviewed by Williams, 2008). Preterm infants often exhibit transient low levels of thyroid hormones (hypothyroxinemia) (Rooman et al., 1996), which is thought to be due to immaturity of the hypothalamic-pituitary-thyroid axis. Long-term follow up of very preterm and/or very low birth weight infants revealed that neurologic dysfunction at age 5, and school failure at age nine, significantly correlated with lower neonatal thyroxine levels (Den Ouden et al., 1996). This result has been challenged by another cohort study in which thyroxine levels in the first 6 weeks of life were correlated with cognitive outcome at 7 years of age, and in this study higher thyroxine exposure was associated with poorer cognitive outcome (Scratch et al., 2014). Consequently, thyroxine substitution therapy was proposed to improve neurodevelopmental outcomes in this vulnerable population. Multiple clinical trials have been conducted, however a systematic review failed to find any significant difference in neonatal mortality, morbidity or neurodevelopmental outcomes in preterm infants who received thyroid hormones compared to controls (Osborn and Hunt, 2007). Since then, additional studies have been completed (Suzumura et al., 2011; Ng et al., 2014; Van Wassenaer-Leemhuis et al., 2014), however only Suzumura et al., found any neurodevelopmental benefit of thyroxine supplementation in preterm infants.

The failure of thyroxine substitution therapy to provide neurological benefit at clinical trial may be related to a lack of monocarboxylate transporter 8 (MCT8, also known as SLC16A2) which is required for transport of thyroid hormones across the cell membrane for its action and metabolism (Friesema et al., 2003). MCT8 expression has been shown to decrease following lipopolysaccharide (LPS)-induced systemic inflammation (Wittmann et al., 2015) and severe intrauterine growth restriction (Chan et al., 2014). It is feasible that preterm infants may similarly exhibit a lack of MCT8, thus may be unresponsive to thyroxine substitution. Future preclinical studies should examine whether up-regulation of MCT8 could improve response to thyroxine, and/or investigate use of a thyroxine hormone analog that does not require MCT8 to cross the plasma membrane.

Nevertheless, two new clinical trials of thyroxine for preterm infants have been registered on clinicaltrials.gov, both yet to start recruiting. The first is a Phase 3 study of 1,224 preterm infants (<28 weeks') randomized to receive either continuous infusion of thyroxine plus oral potassium iodide, or placebo, for 42 days (NCT02103998). The primary outcome for this trial is a composite endpoint of cerebral palsy or Bayley score <85 at 36 months' corrected age. The second is a Phase 2 trial which aims to recruit 100 preterm infants (<28 weeks') with a grade 3–4 IVH to determine whether thyroxine treatment can improve brain structure measured using MRI (NCT03390530). Secondary outcomes include infant mortality and neurodevelopmental impairment at 2 years of age.

#### Minocycline

Minocycline is a semi-synthetic broad-spectrum antibiotic that has been in commercial use for nearly 50 years. It is lipid soluble and can cross the BBB, making it an attractive drug for neurological application. Due to its ability to inhibit microglial activation, minocycline has demonstrated efficacy in various models of adult neurological conditions such as stroke, MS, Parkinson's disease and ALS (Yong et al., 2004; see reviews by Garrido-Mesa et al., 2013). Studies have also shown that minocycline can improve outcomes following perinatal brain injury. For example, minocycline administered 12 h before, and then periodically after LPS exposure, protected against white matter injury, oligodendrocyte loss and abnormal neurobehavioral performance in the neonatal rat (Fan et al., 2005). Similarly, in a preterm rodent model, post-HI treatment with minocycline abolished neuroinflammation and white matter injury (Carty et al., 2008). Notably however, other studies have shown that minocycline actually worsened brain injury in neonatal models (Tsuji et al., 2004; Strahan et al., 2017), although this effect was alleviated when minocycline was administered at a lower dose or later time-point (Strahan et al., 2017). Clearly, dose and timing is very important, and the use of minocycline in pediatric patients warrants careful consideration.

There have been numerous clinical trials conducted of minocycline for various neurological conditions with mixed results. Whilst a 2010 futility study concluded that further investigation of minocycline for Huntington's disease was not warranted (Huntington Study Group Domino Investigators, 2010), data from a recent systematic review indicated efficacy of minocycline in acute stroke patients (Malhotra et al., 2018). Similarly, in a Phase 2 trial for Parkinson's disease, minocycline could not be rejected as futile based on the threshold of a 30% reduction in UPDRS progression (Ninds Net-Pd Investigators., 2006), though no Phase 3 clinical trial has yet been launched. Results are also pending from a dose-escalation study of minocycline for TBI (NCT01058395). Worryingly however, is the finding from a multicenter, randomized Phase 3 trial, that minocycline had a harmful effect on patients with ALS (Gordon et al., 2007). Thus, trials of minocycline, and indeed other neuroprotective agents, should proceed with caution.

#### Epidermal Growth Factor (EGF)

Epidermal growth factor (EGF), together with its receptor EGFR, is a key mediator of neural progenitor cell proliferation, migration and differentiation (reviewed by Galvez-Contreras et al., 2013). In preclinical studies, overexpression of EGFR shifted neural progenitor cells from a non-migratory- to a migratory-phenotype (Aguirre et al., 2005), and EGF stimulated *in vitro* neural progenitor cell oligodendrogenesis (Aguirre and Gallo, 2007). Importantly, EGFR overexpression enhanced proliferation of neural progenitors and their migration to the lesion following lysolecithin-induced focal demyelination in the mouse brain (Aguirre et al., 2007). This led to accelerated and more extensive remyelination, with more rapid functional recovery (Aguirre et al., 2007). Similarly, in a mouse model of preterm hypoxic brain injury, EGFR overexpression or intranasal administration of heparin-binding EGF decreased oligodendrocyte death, promoted oligodendrocyte-progenitor maturation, and led to improved behavioral recovery of mice assessed using various white matter-dependent sensori-motor tests (Scafidi et al., 2014). Moreover, in rabbits with IVH, intracerebroventricular injection of recombinant human EGF significantly increased myelination, promoted proliferation and maturation of oligodendrocyte-progenitors, and enhanced neurobehavioral recovery (Vinukonda et al., 2016). Thus, EGF is an exciting potential neuroprotective therapeutic.

#### Diazoxide

Diazoxide is a potassium channel activator that is used as a vasodilator in the treatment of acute hypertension, and to counter hypoglycemia caused by congenital hyperinsulinism in infants. The neuroprotective potential of diazoxide following ischemia/reperfusion injury was first investigated nearly 20 years ago (Domoki et al., 1999). Despite positive findings, the utilization of diazoxide for reducing neuronal injury in the newborn has been slow to progress. Nevertheless, in a series of *in vitro* and *in vivo* experiments, Fogal et al., demonstrated that diazoxide stimulated proliferation of cultured oligodendrocyte-progenitor cells, enhanced myelination of cerebellar-slice fibers, and prevented hypoxia-induced ventriculomegaly and hypomyelination (Fogal et al., 2010). In a follow-up mechanistic study, the authors' showed that diazoxide actually enhanced maturation of oligodendrocytes, rather than increasing oligodendrocyte proliferation, as their previous *in vitro* studies suggested (Zhu et al., 2014). Given the well-established safety profile of diazoxide, and accumulating efficacy data in stroke, further preclinical studies, in other models of perinatal brain injury, are warranted.

#### Nanoparticle-Targeted Drug Delivery

Nanoparticles for targeted drug delivery are emerging as a promising strategy for the treatment of a range of disorders. Nanoparticle delivery can improve drug bioavailability and targeting, particularly across the BBB. Dendrimers are nanoparticles that have a branching, tree-like structure with a high density of tailorable surface functional groups. Systemically administered hydroxyl-terminated polyamidoamine dendrimers crossed the BBB to localize in activated microglia and astrocytes in the brain of newborn rabbits with cerebral palsy, but not healthy controls (Kannan et al., 2012). Moreover, administration of dendrimers conjugated with the anti-oxidant and anti-inflammatory agent N-acetyl-L-cysteine (NAC; D-NAC), suppressed neuroinflammation and led to a dramatic improvement in motor function. NAC has long been used clinically for acetaminophen poisoning, and has been shown to be an effective treatment for a multitude of psychiatric and neurological conditions including autism, Alzheimer's disease, addiction, schizophrenia, and progressive myoclonic epilepsy (reviewed by Deepmala et al., 2015).

In a mouse model of ischemia-induced neonatal white matter injury, administration of D-NAC suppressed the “detrimental” pro-inflammatory response and increased myelination in regions of white matter injury, indicative of functional recovery (Nance et al., 2015). Furthermore, in a mouse model of perinatal HIE, systemically delivered D-NAC targeted all three types of neural cells involved in brain injury after HI (microglia, neurons, and astrocytes), but only in injured tissue (Nemeth et al., 2017). Importantly, uptake of D-NAC was not significantly altered by exposure to therapeutic hypothermia, suggesting that D-NAC can be used as a combinatorial therapy for the clinical management of HIE (Nemeth et al., 2017). Interestingly, using a mouse model of intrauterine inflammation Lei et al., demonstrated that a single dose of D-NAC, maternally delivered, significantly reduced the rate of preterm birth in pregnant dams exposed to LPS (Lei et al., 2017). Maternal D-NAC treatment altered the placental immune profile, decreased placental T-cell infiltration, and reduced microglial activation. Moreover, D-NAC significantly improved the neuromotor outcomes of LPS-exposed pups (Lei et al., 2017). Whilst the concept of NAC for the prevention of preterm birth is not in itself novel, the high effective dose required for free NAC can induce side-effects of nausea, vomiting, stomatitis, and fever. This publication demonstrates for the first time that low dose NAC delivered via dendrimer-conjugation is effective in reducing preterm birth. Thus, it is feasible that maternally administered D-NAC could be a well-tolerated prophylactic therapy for preterm birth.

Similarly, Chinese researchers have developed a novel catalase-containing nanoparticle that improved outcomes in a rodent stroke model (Zhang et al., 2017a). Catalase is an anti-oxidant and promising neuroprotectant, however its clinical application is hindered by its rapid degradation, immunogenicity and inability to cross the BBB. By modifying catalase containing nanoparticles by (a) cross-linking the polymer to increase drug stability, and (b) adding a peptide tag that is recognized and taken up by infiltrating neutrophils, catalase could effectively piggy-back across the BBB to the target site in the brain (Zhang et al., 2017a). Accordingly, nanoparticle delivery of catalase significantly reduced the infarct volume, mediated by reduced apoptosis, in a mouse model of ischemia/reperfusion injury (Zhang et al., 2017a). It is hypothesized that this technology could be beneficial in any neurological condition characterized by neuroinflammation, including preterm brain injury.

#### Gene Therapy

Gene therapy is the delivery of DNA as a drug to treat disease; the idea being that the transferred DNA would be stably overexpressed as a protein to provide therapeutic benefit. Gene therapy is being actively studied for a range of adult neurological conditions including Alzheimer's disease, ALS and stroke (reviewed in Choong et al., 2016). In contrast, few studies have examined gene therapy for perinatal brain injury. One example however is the overexpression of brain-derived neurotrophic factor (BDNF) to protect the newborn mouse brain from excitotoxic insult. BDNF has been shown to protect the newborn mouse brain from periventricular white matter lesions (Husson et al., 2005). However, since these lesions progress over several days to weeks, the most effective BDNF therapy should involve prolonged/continual administration. Using the same mouse model of neonatal excitotoxic challenge, Bemelmans et al., demonstrated that lentiviral-mediated gene transfer of BDNF to the newborn mouse brain is feasible and affords significant neuroprotection against excitotoxic insult (Bemelmans et al., 2006). Despite these encouraging findings, no published follow-up study was found during the literature search for this review.

#### Emergent Combinatorial Therapies

With the discovery of new, efficacious therapies for neuroprotection and repair of the injured preterm brain, we will undoubtedly see the emergence of combinatorial therapies in an attempt to maximize neurodevelopmental outcome. Some emergent combinatorial therapies are already being tested for various neurological conditions in the clinic, e.g., administration of allogeneic UCB plus EPO in three adults with TBI (Min et al., 2013). Moreover, besides the previously mentioned trial for children with cerebral palsy (NCT02866331), UCB plus G-CSF has been used for stroke patients (Shin and Cho, 2016), and adults with either a brain injury, cerebral palsy, Parkinson's disease or ALS (NCT02236065). Interestingly, EPO together with G-CSF has been proposed for adults with neurological diseases (NCT02018406), though the status of this trial is unknown. Unsurprisingly, various other combination therapies are being investigated at the preclinical stage. Recently, genetically engineered UCB mononuclear cells were used to deliver triple gene therapy in a rodent model of stroke (Sokolov et al., 2018). Treatment with minocycline followed by NAC is showing encouraging results for TBI (Sangobowale et al., 2018), and Jantzie et al., is exploring postnatal administration of EPO and melatonin in a rat model of perinatal brain injury caused by extreme preterm birth (Jantzie et al., 2018).

### Neuroplasticity-Inducing Rehabilitation

#### Neuroplasticity

The brain's capacity to recover after injury is what makes early intervention scientifically possible. Neuroplasticity encompasses the ability of the brain to organize/reorganize its circuitry and produce new cells (neurogenesis) and connections (synaptogenesis) (Johnston, 2009). Environmental stimulation (early intervention) protects neurons and promotes the secretion of growth factors in the brain (Vaccarino and Ment, 2004). Neuroplasticity occurs at all ages however the developing brain has greater capacity for change. Both genetic and environmental factors play important roles in neuroplasticity during development.

#### Critical Periods and Timing

The preclinical work of Kolb and others show that critical periods exist during development in which the brain's capacity to reorganize in response to injury is enhanced while at other times neuroplastic mechanisms can be detrimental (Kolb et al., 2011). A suite of experiments involving various drug therapies and neurotrophic agents has demonstrated the ability of the developing brain to develop new neural networks when treatments were strategically introduced during known critical periods.

Kolb et al. describe three types of neuroplasticity: experience independent (largely genetically determined but modified by both internal and external events), experience dependent plasticity which lasts a lifetime, and experience expectant plasticity (Kolb et al., 2017). This latter type is characterized by a critical period that is moderated by a balance of inhibitory and excitatory inputs that may be chemically modifiable, thus enabling a widening of the critical period.

Environmental enrichment is the most commonly used method of inducing neuroplasticity in animal studies. The harnessing of activity-dependent mechanisms by enriching the housing and social environments of experimental animals has demonstrated improved outcomes after injury in both adult and infant models (Nithianantharajah and Hannan, 2006). The use of enriched environments for animals with perinatal brain injury produces structural brain changes including increased brain volumes as well as improved cognitive, motor and behavioral skill acquisition. Tactile stimulation, or sensory enrichment has been shown in numerous studies to improve cognitive, motor and visual function (Sale et al., 2009; Kolb et al., 2017).

Although activity-dependent plasticity is a lifelong mechanism, the timing of experience and training post injury seems important. A cat model of cerebral palsy showed that early stimulation of the corticospinal tract using forelimb training resulted in functional skill acquisition as well as connectivity, whereas animals who received delayed training did not develop functional use of the affected limb (Friel et al., 2012). Studies in adult stroke patients have also indicated that training close to the time of the infarct leads to better functional skills and structural brain changes (Kleim and Jones, 2008).

#### Principles of Experience Dependent Neuroplasticity

Kleim and Jones describe activity-dependent neuroplasticity principles that should be applied to rehabilitation programs in humans with brain damage, stressing the importance of active engagement of neural circuits to avoid degradation and enhance function (Kleim and Jones, 2008). Although primarily focused on adult and animal studies these principles (task specificity, repetition, salience, timing, and intensity) also apply to infants with brain damage who need to learn for the first time, rather than re-learn, how to move, think, and communicate. Thus the principles of neuroplasticity along with environmental enrichment strategies should be applied in infant early intervention, with reference to the child's context—most importantly the parent-child relationship. Attachment to a primary caregiver is paramount for early brain development as studies of institutionalized children attest (Fox et al., 2011). Early intervention programs for infants with brain injuries should support not only the parent (Benzies et al., 2013), who are at higher risk of poor mental health (Whittingham et al., 2014) but also provide support to the child-parent relationship in order to maximize outcomes.

Measuring neuroplasticity using advanced imaging techniques is possible in adults post-stroke (Cramer et al., 2011), and increasingly advocated for children with cerebral palsy (Reid et al., 2017). Imaging of cortical thickness and gray matter can identify neuroplastic change (Reid et al., 2015) and has been conducted in very young children with unilateral cerebral palsy post constraint induced movement therapy (Sterling et al., 2013). Transcranial magnetic stimulation can also be used to measure cortical reorganization and is currently being trialed in infants post perinatal stroke (Chen et al., 2017). However, ethical dilemmas exist for this age group since conducting MRI for research purposes require anesthetics to guarantee useable images.

#### Neuroplasticity-Inducing Rehabilitation Trials

A multitude of early intervention trials have been conducted in cohorts of preterm infants. These interventions may be delivered during the NICU stay (Ustad et al., 2016) or commence post discharge (Spittle et al., 2010). Some intervention studies start during NICU admission and continue for a short period after discharge (Dusing et al., 2015, 2018). Since it is clear that prematurity significantly impacts all developmental domains, in most instances study inclusion is based on gestational age or birthweight. A meta-analysis of controlled trials (random and quasi randomized) demonstrates that cognitive outcomes are significantly improved through early intervention, with effects lasting until school-age (Spittle et al., 2015). Motor outcomes however were only marginally improved in these studies and the clinical significance of the improvement is not clear. This systematic review included any early developmental intervention programs that aimed to improve cognitive or motor outcomes initiated within 12 months post-term age. Types of interventions included physiotherapy, occupational therapy, neurodevelopmental therapy, parent-infant relationship enhancement, infant stimulation, and early education intervention. Thus the included interventions are heterogeneous in terms of content, dose and outcome measurement (Spittle et al., 2015), making it difficult to draw out the key ingredients for beneficial effects. Most approaches focus on training parents to recognize and respond to their infant's cues as well as providing information and guidance to support the infant to meet developmental milestones. Some early intervention approaches are more child focused and delivered by a physiotherapist or occupational therapist (Spittle et al., 2015).

Very few studies target preterm infants with brain injuries and in fact many studies specifically exclude preterm infants with imaging abnormalities. The two small RCTs that specifically targeted preterm infants with cerebral injuries did not find statistically significant between group differences in either motor or cognitive outcomes (Nelson et al., 2001; Ohgi et al., 2004), although short term neurobehavioral benefits were found in one study (Ohgi et al., 2004).

##### Environmental enrichment

Whilst the impact of prematurity on cerebral brain development has been extensively studied, the impact of the NICU admission itself on the brain is less certain. Painful or stressful stimuli and deprivation can have profound negative effects on brain development. It is difficult to disentangle prematurity and its associated co-morbidities from the life-saving therapies, but also the various “threats” that admission into an intensive care environment provides. The magnitude of these threats is being more accurately quantified, and we now know that the premature infant goes into an environment of light for most of the 24 h cycle (Rodriguez and Pattini, 2016), a relatively noisy environment (Shoemark et al., 2016), and a situation in which painful procedures e.g., heel lances, are common (Brummelte et al., 2012). Reproduction of the *in utero* environment, with low-level sensory input and safety, is not currently possible, however clinician scientists are now beginning to investigate the potential benefit of “normalizing” the environment for the preterm infants, for example by promoting appropriate circadian sleep-wake rhythm cycles and protecting the infant from sensory overload. Length of stay in the NICU is rarely controlled for as a confounder in RCT's, but it is easily quantified and is often used as a short term outcome in trials of neonatal interventions—with reduced length of stay considered as a significant positive outcome, both for the baby, the family, and the health service economist.

Enriching the newborn care environment via the Newborn Individualized Care and Assessment Program (NIDCAP) is an intervention that has been widely adopted (reviewed in Moody et al., 2017). NIDCAP aims to provide an environment with minimal stress that adapts to the needs of the infant through identification and response to infant cues. Though some early trials showed improved brain function and structure in low-risk preterm infants (Als et al., 2004), subsequent studies have generally not reproduced these findings, and meta-analyses do not show significant gains from NIDCAP in the short or long term (Ohlsson and Jacobs, 2013). To date NIDCAP does not appear to prevent or ameliorate brain injury in preterm infants, nor does it do harm. Studies of infant massage (sensory enrichment) have shown positive results in a small sample of preterm infants (Guzzetta et al., 2009), as has music therapy (Lubetzky et al., 2010).

Although early intervention aims to harness naturally occurring neuroplastic mechanisms such as experience-dependent plasticity, a number of interventions that have been studied have involved the infant largely in a passive capacity in a clinic-based environment (Weindling et al., 1996).

Systematic review evidence indicates that a small effect on motor outcomes is gained for infants 0–2 years with brain injuries, when interventions are applied that enrich at least one aspect of the environment (social, motor, sensory, or cognitive) (Morgan et al., 2013). Since this review, two further studies of preterm infants (with and without brain damage) (Dusing et al., 2015, 2018) have shown early promising results in early problem solving skills. “SPEEDI” (Supporting Play Exploration and Early Developmental Intervention) is an intervention where parents are coached and supported to promote daily movement opportunities and environmental enrichment strategies with their infant. Larger trials are underway to explore the importance of timing of the intervention (NCT03518736).

##### Training motor skills

Traditional physiotherapy for cerebral palsy has been largely a passive experience for the child, however systematic review evidence favors approaches that are goal directed and involve repetitive practice of specific and functional tasks (Novak et al., 2013). Recent systematic reviews of early intervention (from birth to 24 months) for infants with cerebral palsy have not only revealed the lack of rigorous trial data available but also that traditional early intervention is largely ineffective for infants with cerebral palsy (Morgan et al., 2016a). The majority of studies identified in this review did not demonstrate any motor gains for infants with cerebral palsy. However, only half of the included trials began early intervention prior to 6 months of age. Delayed definitive diagnosis of cerebral palsy, after 12 months, has typically meant that a mixed group of “at risk” infants are recruited, leading to underpowered trials. Alternatively, waiting for a confirmed diagnosis prior to recruitment means that infants with cerebral palsy don't receive intervention until the second year of life.

An important study from 1988 randomized 48 infants with spastic diplegia, most of whom were preterm, to receive either a home-based active enrichment intervention (“Learning Games”) or Neurodevelopmental Therapy, the most commonly applied physiotherapy intervention (Palmer et al., 1988). Infants randomized to the enrichment intervention were significantly more advanced in motor and cognitive skills after 6 months of intervention as measured using the Bayley.

Evidence for early interventions that involve active motor skill training has been growing slowly over the last 4 years. Two RCTs of GAME (Goals Activity Motor Enrichment; Morgan et al., 2014) intervention in infants aged 3–6 months with or at high risk of cerebral palsy demonstrated improved motor (Morgan et al., 2015, 2016b) and cognitive skills (Morgan et al., 2016b) after a minimum of 3 months intervention, when compared to standard care. GAME is a motor learning intervention that coaches and supports parents to train motor and cognitive skills through play, in an enriched environment. GAME is always delivered in the family home with practice carried out by parents throughout the week between face to face therapy sessions. A large multicenter single blind randomized controlled trial (*n* = 300) of GAME vs. standard care is currently being undertaken (ACTRN12617000006347).

About 20–30% of preterm infants with cerebral palsy will be diagnosed with unilateral cerebral palsy (Himpens et al., 2008; ACPR Group, 2018). High quality evidence exists for the use of constraint induced movement therapy (CIMT) in children with unilateral cerebral palsy (Novak et al., 2013) and there is early evidence to support the use of a modified version of CIMT for infants. A retrospective study of 72 infants with unilateral cerebral palsy confirmed that children who had received CIMT as an infant (baby-CIMT) were significantly more likely to have superior hand function at age two than those who did not, even when controlling for the type of lesion (Nordstrand et al., 2015). A recently published RCT (*n* = 37) confirmed that infants with unilateral cerebral palsy had better function of their affected hand after 36 h of baby-CIMT than infants who had received the same dose of infant massage (Eliasson et al., 2018). Bimanual therapy, a training intervention that targets using both hands together, is equally effective as CIMT at a matched dose in older children with hemiplegia however trials in infants are not yet published (Sakzewski et al., 2014). A randomized trial comparing baby CIMT with bimanual training is currently underway (Boyd et al., 2017).

While the evidence for early habilitation interventions in infants with cerebral palsy is small it is steadily growing evidenced by the large number of registered clinical trials underway and an increasing focus on earlier detection (Byrne et al., 2017; Novak et al., 2017). Further research is required regarding interventions that can be applied very early (during the NICU) in preterm infants at high risk of cerebral palsy since motor and sensory pathways are still developing at this point. Ideally, combining pharmacological and/or cell based therapies with habilitation interventions to drive experience-dependent plasticity, appears to be the approach that should be targeted by those working with preterm infants with cerebral palsy.

## Potential Future Advances: Biomarkers of Preterm Brain Injury

An early diagnosis of cerebral palsy at 12-weeks of age corrected is a major step forward, but still falls short of the need to accurately diagnose brain injury and predict the likelihood of disability within hours of injury, so as to instigate novel and conventional protective treatments, such as therapeutic hypothermia in the case of HIE. The race is now on to identify reliable biomarkers of brain injury.

Blood biomarkers are under intense investigation, because the BBB “opens” after injury (Anblagan et al., 2016). This opening is mediated by a number of factors, including disruption of the tight-junction complexes connecting brain endothelial cells, increased production of inflammatory mediators resulting in infiltration of immune cells into the injured site. A permeabilized BBB enables by-products of the injury cascade to be released into the circulatory system (Graham et al., 2018). Several candidate biomarkers have been identified and have been thoroughly appraised in a systematic review (Graham et al., 2018). Candidate biomarkers include: Glial Fibrillary Acidic Protein (GFAP); Neuron-Specific Enolase (NSE); S100B; Ubiquitin Carboxy-Terminal Hydrolase L1 Protein (UCHL1); Cleaved Tau (C-TAU); Microtubule-Associated Protein 2 (MAP2); Myelin Basic Protein (MBP); Spectrin Breakdown Products (SBDP); Brain-Derived Neurotrophic Factor (BDNF); Activin A; Matrix Metalloproteinase-9 (MMP-9); Vascular Endothelial Growth Factor (VEGF); Platelet Derived Growth Factor Receptor b (PDGFRb); Thrombospondin-1 (TSP-1); Tumor Necrosis Factor Alpha (TNF-a); Granulocyte Colony Stimulating Factor (G-CSF); MicroRNA (miRNA); and Exosomes (Graham et al., 2018). These proteins, neurotrophins, factors, inflammatory and metabolic markers are only released peripherally after injury, and are therefore being studied as indicators of the presence of brain injury. Importantly, it is likely that a panel/s of biomarkers will be most useful for identifying infants with neurological injury, and a combination of markers, in addition to existing tools, would provide the greatest diagnostic sensitivity (Yokobori et al., 2013).

In addition to blood biomarkers, neuroimaging techniques including ultrasonography, MRI and electroencephalography (EEG) are crucial tools in the diagnosis and prediction of prognosis following preterm brain injury (Jin et al., 2015). Though current techniques have distinct advantages and disadvantages (e.g., cost, accessibility, sensitivity, need for general anesthetic during MRI), development of advanced techniques such as volumetric MRI, diffusion tensor imaging, metabolic imaging (MR spectroscopy), functional ultrasound (Deffieux et al., 2018) and amplitude-integrated EEG, are transforming the neuroimaging field. Advanced MRI techniques as well as the “Baby Connectome Project” (Howell et al., 2019) are likely to result in the development of improved diagnostic and prognostic markers in addition to novel outcome measures for quantifying neuroplasticity change from intervention.

Importantly, it has been proposed that a combination or panel of biomarkers may provide clues about the type of injury, allowing identification of new treatment paradigms that address the underlying cellular damage mechanisms. In addition, biomarkers may enable clinical triage of combinatory treatments and therefore they offer great promise to the field (Graham et al., 2018).

## Conclusions

Preterm birth is a significant cause of morbidity and mortality for infants, and remains a significant risk factor for cerebral palsy. Encouragingly, there are numerous emerging biological, pharmacological, and rehabilitation interventions for the prevention, minimization, and even reversal of preterm brain injury. Many of these therapies are showing promising signs of efficacy in early-phase clinical trials and preclinical studies. However, as new interventions emerge, so arise various controversies surrounding their implementation, which need to be solved before they can gain acceptance and be widely implemented in the clinic. Finally, the development of reliable biomarkers of preterm brain injury, will enable better detection, diagnosis and prediction of long-term outcomes, which will undoubtedly lead to more efficacious, better-targeted therapies, with improved outcomes for infants born preterm.

## Author Contributions

MF-E, CM, and IN: conceived the project. MF-E, CM, RH, and IN: drafted the article. All authors were involved in editing and approval of the final manuscript.

### Conflict of Interest Statement

The authors declare that the research was conducted in the absence of any commercial or financial relationships that could be construed as a potential conflict of interest.
